# Complications following upfront pancreatectomy with venous resection do not compromise adjuvant chemotherapy delivery and survival in pancreatic cancer

**DOI:** 10.1007/s00423-025-03933-z

**Published:** 2025-11-27

**Authors:** Giampaolo Perri, Samuele Grandi, Muyue Liu, Riccardo Pellegrini, Jianzhen Lin, Nicola Canitano, Riccardo Guastella, Zipeng Lu, Domenico Bassi, Umberto Cillo, Kuirong Jiang, Giovanni Marchegiani

**Affiliations:** 1 Hepato-pancreato-biliary and Liver Transplant Surgery Unit, Department of Surgical, Oncological and Gastroenterological Sciences (DiSCOG), University of Padua, Via Giustiniani 2, Padua, 35128 Italy; 2 Pancreas Center, The First Affiliated Hospital of Nanjing Medical University, Institute of Pancreas, Nanjing Medical University, Nanjing, China

**Keywords:** Pancreatectomy, Vein, Resection, Cancer, Chemotherapy

## Abstract

**Purpose:**

The combined burden of vascular resections and pancreas-specific complications may preclude or delay adjuvant chemotherapy and impair survival. We evaluated the effect of complications on adjuvant therapy delivery and survival after upfront pancreatectomy with venous resection (PVR).

**Methods:**

Patients undergoing upfront PVR were retrieved from a prospectively maintained database at two high-volume Institutions. The incidence and severity of complications were correlated with administration of adjuvant chemotherapy and overall survival.

**Results:**

Overall, 280 patients underwent upfront PVR. 75% (*N* = 210) underwent pancreatoduodenectomy (PD), 15% (*N* = 41) distal pancreatectomy (DP), and 10% (*N* = 29) total pancreatectomy (TP). Major morbidity occurred in 34% (*N* = 96), with 4% (*N* = 12) 90-day mortality. Overall rates of POPF, PPH, and DGE were 22%, 15%, and 18%, respectively. Mortality was higher in Type IV venous resections (14%, *p* = 0.028). DP was associated with higher morbidity but similar mortality compared to PD and TP. The only factor independently associated with adjuvant chemotherapy delivery, administered in 196 (70%), was ASA score < 3 (*p* = 0.003). Factors independently associated to worse OS were age > 75 years, TP, pT > 2, pN2, and lack of adjuvant chemotherapy delivery.

**Conclusions:**

Upfront PVR has an acceptable risk profile and oncologic outcomes when adjuvant chemotherapy is administered. Survival and the delivery of adjuvant therapy do not appear to be negatively affected by complications.

**Supplementary Information:**

The online version contains supplementary material available at 10.1007/s00423-025-03933-z.

## Introduction

Pancreatic ductal adenocarcinoma (PDAC) is a highly aggressive malignancy often diagnosed at an advanced stage, with only 15% of cases found to be resectable at the time of detection [1]. The best treatment outcomes are currently achieved only through a combination of surgical resection and chemotherapy. This multimodal approach offers the greatest chance for prolonged survival, highlighting the need for improved treatment strategies and multidisciplinary care. A significant proportion of PDAC exhibit a certain degree of vascular involvement, which not only presents a greater surgical challenge for resection but also reflects the tumour’s tendency towards an aggressive local behaviour. Therefore, vascular resections play an essential role in achieving radicality and represent nowadays a key element in the pancreas surgeon toolbox. Their safety and feasibility are well established, particularly at high-volume centres with substantial expertise [2].

The role of the neoadjuvant chemotherapy is widely recognized as pivotal for tumors with vascular involvement, namely those classified as borderline resectable and locally advanced. However, for tumors deemed primarily resectable, the optimal strategy remains unclear [3]. Current guidelines still recommend upfront resection for resectable stage, unless neoadjuvant therapy is assessed within clinical trials [4]. Minor venous involvements, defined as less than 180° vessel contact without contour irregularity, are considered resectable, therefore vascular resections may still be necessary in these cases. This aspect might generate controversies, as vascular resections in this setting might significantly add to the burden of postoperative complications, which may result in preventing patients from receiving adjuvant chemotherapy and ultimately impair long-term survival [5]. In fact, adjuvant chemotherapy is often not administered to all eligible patients postoperatively, primarily due to postoperative complications that contraindicate further treatment.

The aim of the present study is to investigate the impact of postoperative complications after upfront pancreatectomy with venous resection (PVR) for PDAC, focusing on their potential detrimental effect on adjuvant therapy delivery and OS.

## Materials and methods

This study was a retrospective, bicentric analysis of prospectively maintained databases including data from patients treated at the Hepato-Pancreato-Biliary and Liver Transplant Surgery Unit of Padova University Hospital (Italy) and at the Pancreas Center of Nanjing University Hospital (China). The study complied with the regulations of the Institutional Review Boards of both University Hospitals (Approval numbers: 6281/AO/25 - AOP3758).

### Study population and definitions

All patients who underwent upfront pancreatectomy with PVR for PDAC between 2015 and 2023 were included. Inclusion criteria were venous resection, histologically confirmed PDAC, and age ≥ 18 years. Exclusion criteria consisted of prior neoadjuvant chemotherapy. Pancreatic resections were categorized as pancreatoduodenectomy (PD), distal pancreatectomy (DP), or total pancreatectomy (TP). Venous resections were classified according to the ISGPS classification [6], which categorizes four types based on tangential (Type I and II) or segmental (Type III and IV) resection. In details they were defined as Type I = tangential w/primary closure; Type II = tangential w/peritoneal or prosthetic patch; Type III = termino-terminal w/primary anastomosis; and Type IV=) termino-terminal w/interposition graft. The intraoperative venous reconstruction choice was determined by highly experienced surgeons based on tumor location, extent and degree of vascular involvement, preference, and graft availability. Pancreas-specific complications — including postoperative pancreatic fistula (POPF) [7], post-pancreatectomy hemorrhage (PPH) [8], and delayed gastric emptying (DGE) [9]— were reported in accordance with the ISGPS grading system. Major morbidity was defined as Clavien-Dindo [10] grade ≥ 3 complications. Tumor staging was reported according to the AJCC [11], and R1 resection was defined as the presence of viable tumor cells within 1 mm of the resection margin [12]. After resection, individual patient and tumor characteristics determined the need for and type of postoperative therapy. This group comprised individuals who began adjuvant treatment and received at least one cycle. The specific reasons for the lack of access to chemotherapy were not recorded in the prospectively collected database. Prophylactic dose of heparin was routinely administrated postoperatively for 30 days after surgery, except for graft interposition where a therapeutic dose was used. After surgery, patients were typically evaluated every 3 to 4 months at first and then every 6 months with cross-sectional imaging, physical examination, and CA 19 − 9 analysis. The primary endpoint of the study was to identify intra- and postoperative factors associated with adjuvant treatment administration and poor overall survival (OS).

### Statistical analysis

Statistical analysis was performed using R 4.2.3 software. Statistical significance was set at a two-tailed P-value < 0.05. Descriptive analysis was reported as median with interquartile range (IQR, 25%−75%) for continuous variables and as frequencies and percentages for categorical variables. Pearson’s chi-square test was used for the comparison of categorical variables and when the frequencies were lower than 5, the Fisher’s test was used. Binomial logistic regression was performed to analyze factors associated with adjuvant therapy administration, and results were presented as Odds Ratio (OR) with 95% confidence intervals (CI). OS was calculated from the date of surgery to the date of death or last follow-up. Kaplan-Meier analysis was used to generate survival curves, and a Cox regression model with Hazard Ratio (HR) and 95% confidence intervals (CI) was applied to identify potential predictors of poor survival.

## Results

A total of 280 consecutive patients underwent upfront PVR and were included in the analysis. Baseline and preoperative characteristics are shown in Table 1. The median age was 65 years (57–72) and 65 (25%) patients were categorized with American Society of Anesthesiology (ASA) grade ≥ 3. The median preoperative CA19-9 level was 166 U/mL (41–497), with the tumor being primarily located in the head of the pancreas (79%). Most patients were radiographically staged as resectable (*N* = 227; 81%) according to NCCN [13].

**Table 1:** Perioperative characteristics of all patients (*N* = 280)

Characteristics	Total, No. (%) (*N* = 280)
Preoperative - demographic	
Age, median (IQR), y	65 (57–72)
Female sex, No. (%)	134 (48)
ASA score ≥ 3, No. (%)	65 (25)
Ca 19.9, median (IQR), UI/L	166 (41–497)
Tumor location, No. (%)	
Head	221 (79)
Body-tail	48 (17)
Multifocal	11 (4)
Radiographic staging (NCCN)	
Resectable	227 (81)
Borderline resectable	53 (19)
Intraoperative - general	
Surgery type, No. (%)	
Pancreatoduodenectomy	210 (75)
Distal pancreatectomy	41 (15)
Total pancreatectomy	29 (10)
Operative time, median (IQR), min	346 (271–404)
Venous resection, No. (%)	
Tangential	70 (25)
Segmental	210 (75)
ISGPS type, No. (%)	
I. (Tangential with primary closure)	49 (17)
II. (Tangential with peritoneal patch)	21 (7)
III. (T-T with primary anastomosis)	189 (68)
IV. (T-T with interposition graft)	21 (7)
Site of anastomosis, No. (%)	
PV-PV	35 (13)
SMV-SMV	87 (31)
PV-SMV	158 (56)
Concomitant arterial resection, No. (%)	15 (5)
Intraoperative heparine (reconstruction phase), No. (%)	51 (18)
Pathology	
Tumor diameter, median (IQR), mm	35 (30–45)
T status, No. (%)	
T1	10 (4)
T2	108 (38)
T3	77 (27)
T4	80 (29)
Missing	5 (2)
Number of harvested lymphnodes, median (IQR)	19 (15–27)
Number of positive lymphnodes, median (IQR)	2 (2)
Lymphnode ratio, median (IQR), %	8 (0–20)
N status, No. (%)	
N0	82 (29)
N1	130 (46)
N2	63 (23)
Missing	5 (2)
AJCC TNM Stage, No. (%)	
IA	5 (2)
IB	25 (9)
IIA	29 (10)
IIB	90 (32)
III	126 (46)
Missing	5 (2)
R status, No. (%)	
R0	122 (44)
R1	158 (56)
Postoperative - general	
POPF grade (if PD/DP), No. (%)	
BL	27 (11)
B	53 (21)
C	3 (1)
PPH grade, No. (%)	
A	12 (4)
B	11 (4)
C	20 (7)
DGE grade, No. (%)	
A	15 (5)
B	13 (5)
C	23 (8)
Biliary leak (if PD/TP), No. (%)	6 (2)
Chyle leak, No. (%)	34 (12)
Enteric leak, No. (%)	2 (1)
Relaparotomy, No. (%)	23 (8)
Major morbidity (Clavien-Dindo ≥ 3), No. (%)	96 (34)
Mortality (30-days), No. (%)	3 (1)
Mortality (90-days/in-hospital), No. (%)	12 (4)
Adjuvant therapy, No. (%)	196 (70)

*BMI* body mass index, *PDAC* pancreatic ductal adenocarcinoma, *PV* portal vein, *SMV* superior mesenteric vein, *PD* pancreatoduodenectomy, *DP* distal pancreatectomy, *TP* total pancreatectomy, *ISGPS* International Study Group on Pancreatic Surgery, *AJCC* American Joint Committee on Cancer, *POPF* postoperative pancreatic fistula, *BL* biochemical leak, *POH* postoperative hyperamylasemia, *PPH* post-pancreatectomy hemorrhage, *DGE* delayed gastric emptying, *ICU* intensive care unit, *POD* postoperative day

### Surgical procedures

Intraoperative characteristics are shown in Table 1. Regarding surgical procedures, 75% (*N* = 210) underwent pancreaticoduodenectomy (PD), 15% (*N* = 41) distal pancreatectomy (DP), and 10% (*N* = 29) total pancreatectomy (TP). The most frequently performed venous resection was end-to-end primary anastomosis (Type III), accounting for 189 cases (68%), followed by tangential resection with primary closure (Type I) in 49 patients (17%). The distribution of the type of venous resection according to the surgical procedure is shown in Supplementary Table 1. There was a significant difference in the distribution of venous resections among different pancreatic resections (*p* = 0.008), as Type III was the most used resection in PD (71%) and TP (65%), while DP had a high rate of Type I resections (39%), together with Type III (49%). Graft interposition (Type IV) was overall utilized in a minority of cases (8%, *N* = 21), but most frequently in TP (14%; *N* = 4). Concomitant arterial resections were performed in 15 (5%) patients. Less than 20% of patients received intraoperative heparin during the reconstruction phase.

### Pathology

Pathology reported a median tumor diameter of 35 mm (30–45), with AJCC stage III being the most frequent (*N* = 126, 46%), followed by stage IIb (*N* = 90, 33%). A positive lymph node status was observed in 193 patients (70%), with N2 in 63 cases (23%). A R1 margin status was present in 56% of patients. Overall, 70% of patients had confirmed venous invasion at pathology. Uncomplete pathological data were present in 5 patients. Pathology data are presented in Table 1.

### Pancreas-specific complications

Data from the entire cohort are reported in Table 1. The overall 30- and 90-day mortality rates were 1% (*N* = 3) and 4% (*N* = 12), respectively. Major surgical complications occurred in 96 (34%) patients, and 23 (8%) required relaparotomy. Regarding pancreatic-specific complications, the overall rates of POPF, PPH, and DGE were 22%, 15%, and 18%, respectively. Regarding POPF, 53 (21%) patients had grade B and 3 (1%) grade C. PPH grade C occurred in 20 (7%) patients, while DGE grade C was observed in 23 (8%) patients. Analysis of pancreas-specific complications revealed different distributions of POPF and PPH based on venous resection types (Supplementary Table 1). Overall, PPH was most frequent in ISGPS type IV (33%, *p* = 0.024), though there was no significant difference in grade C PPH. POPF was observed in 35% and 37% of ISGPS type I and II cases, compared to 18% and 12% of ISGPS type III and IV, respectively (*p* = 0.022). The frequency of all DGE grades was normally distributed (*p* = 0.925). The 90-day mortality was highest in ISGPS type IV (*N* = 3, 14%, *p* = 0.028). Conversely, when considering pancreatic resections (Supplementary Table 2), 49% of DP experienced POPF versus 17% of PD (*p* < 0.001), with higher major morbidity rate in the DP group (*N* = 23, 56%, *p* = 0.006) compared to PD and TP. PPH occurred more frequently in TP (*N* = 9, 31%, *p* = 0.035). No significant differences were observed in DGE or mortality distributions among different pancreatic resection types.

### Adjuvant chemotherapy

Adjuvant chemotherapy was administered to 196 patients (70%). Results are shown in Table 2. Patients who received chemotherapy experienced less frequently PPH (11% vs. 26%, *p* = 0.001) and did not have grade C POPF (*p* = 0.037). Moreover, lower rates of major morbidity (29% vs. 46%, *p* = 0.005) and relaparotomy (5% vs. 17%, *p* < 0.001) were observed in patients undergoing adjuvant therapy. A logistic regression analysis was performed to investigate the factors associated with its administration, and, at multivariable analysis, only ASA < 3 retained significance (*p* = 0.003) as an independent predictor of adjuvant therapy administration (Table 3).

**Table 2:** Type of resections, complications & mortality rates according to adjuvant chemotherapy administration

Events	Total, No. (%) (*N* = 280)	Adjuvant administration, No. (%)		*P*
		No (*N* = 84) (30%)	Yes (*N* = 196) (70%)	
ISGPS venous type I II III IV	49 (18%) 21 (7%) 189 (68%) 21 (7%)	18 (22%) 8 (9%) 50 (60%) 8 (9%)	31 (15%) 13 (7%) 139 (71%) 13 (7%)	0.321
Surgery type, No. (%) PD DP TP	210 (75%) 41 (15%) 29 (10%)	59 (70%) 14 (17%) 11 (13%)	151 (77%) 27 (14%) 18 (9%)	0.457
DGE (yes) Grade C	51 (18%) 23 (8%)	16 (19%) 6 (7%)	35 (17%) 17 (9%)	0.946 0.849
PPH (yes) Grade C	43 (15%) 20 (7%)	22 (26%) 10 (12%)	21 (11%) 10 (5%)	**0.001** 0.076
POPF (yes) Grade: B C	56 (22%) 53 (21%) 3 (1%)	20 (27%) 17 (23%) 3 (4%)	36 (20%) 36 (20%) 0	0.283 0.712 **0.037**
Major morbidity	96 (34%)	39 (46%)	57 (29%)	**0.005**
Relaparotomy	23 (8%)	14 (17%)	9 (5%)	**< 0.001**

*PD* pancreatoduodenectomy, *DP* distal pancreatectomy, *TP* total pancreatectomy, *ISGPS* International Study Group on Pancreatic Surgery, *DGE* delayed gastric emptying, *PPH* post-pancreatectomy hemorrhage, *POPF* postoperative pancreatic fistula

**Table 3:** Univariable and multivariable logistic regression analysis of predictors for adjuvant chemotherapy administration

Predictors	Univariable			Multivariable		
	OR	95% CI for OR	*p*	OR	95% CI for OR	*p*
Age > 75 years	0.38	0.2–0.7	**< 0.001**	0.44	0.2–1.03	0.059
Gender Female	1.16	0.7–1.9	0.566			
ASA < 3	3.18	1.7–5.7	**< 0.001**	2.6	1.4–5.13	**0.003**
Segmental vs. tangential resections Tangential	1.55	0.9–2.7	0.134			
Surgery DP (ref.) vs. PD Total (ref.) vs. PD	0.7 0.6	0.4–1.5 0.3–1.4	0.436 0.278			
‍Concomitant arterial resection yes	1.76	0,5–6.4	0.391			
Margin status R1	1.1	0.6–1.8	0.713			
pT T1-T2 vs. T3-T4	0.8	0.5–1.3	0.386			
Stage ≥ III	1.34	0.8–2.2	0.276			
Relaparotomy yes	0.2	0.1–0.6	**0.002**	0.6	0.2–2	0.400
CR-POPF yes	0.67	0.3–1.2	0.217			
PPH yes	0.33	0.2–0.6	**0.013**	0.6	0.2–1.5	0.305
DGE yes	0.9	0.4–1.7	0.813			
Major morbidity yes	0.46	0.3–0.8	**0.005**	0.5	0.3–1.1	0.096

*PD* pancreatoduodenectomy, *DP* distal pancreatectomy, *TP* total pancreatectomy, *ISGPS* International Study Group on Pancreatic Surgery, *POPF* postoperative pancreatic fistula, *PPH* post-pancreatectomy hemorrhage, *DGE* delayed gastric emptying

### Survival and prognostic factors

**Fig. 1:**
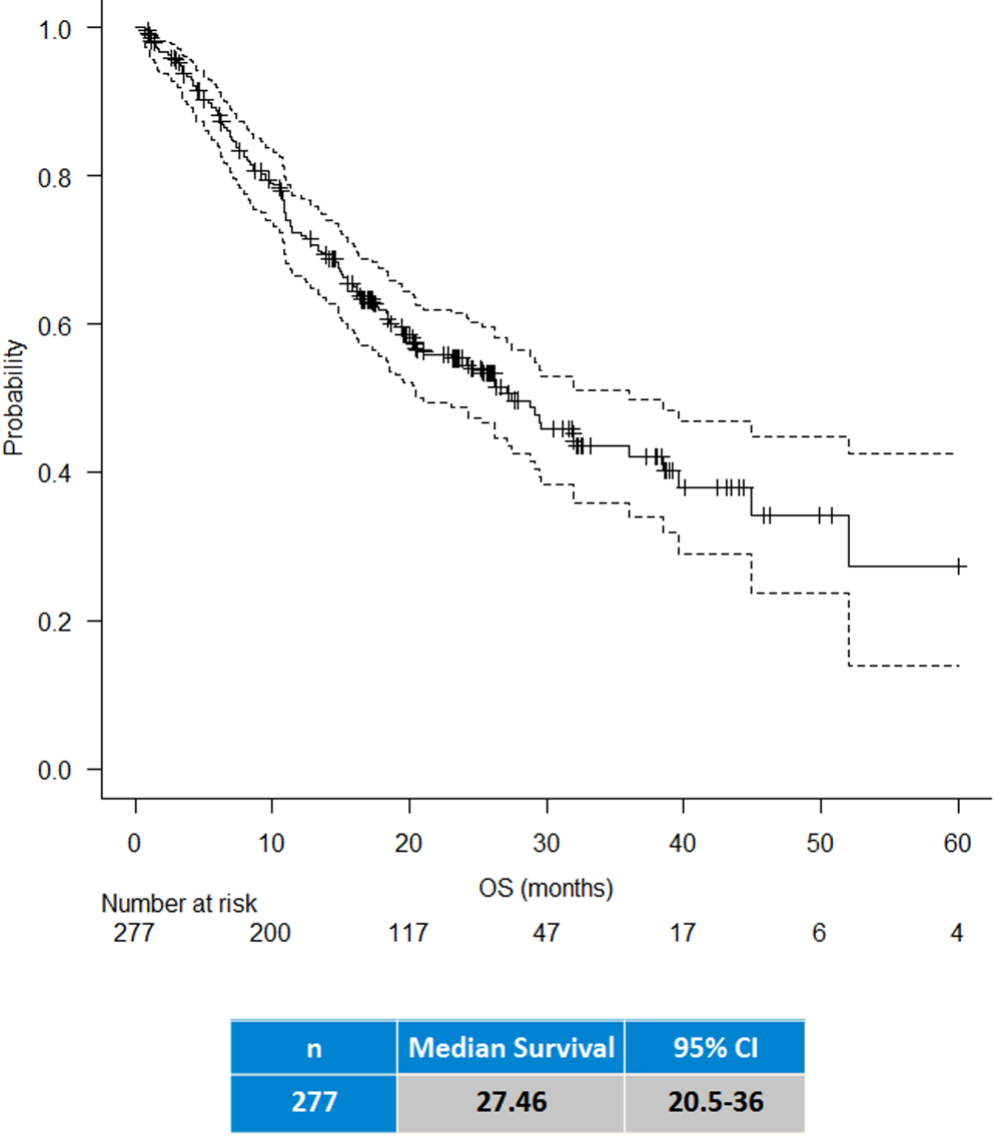
Overall Survival after upfront PVR

**Fig. 2:**
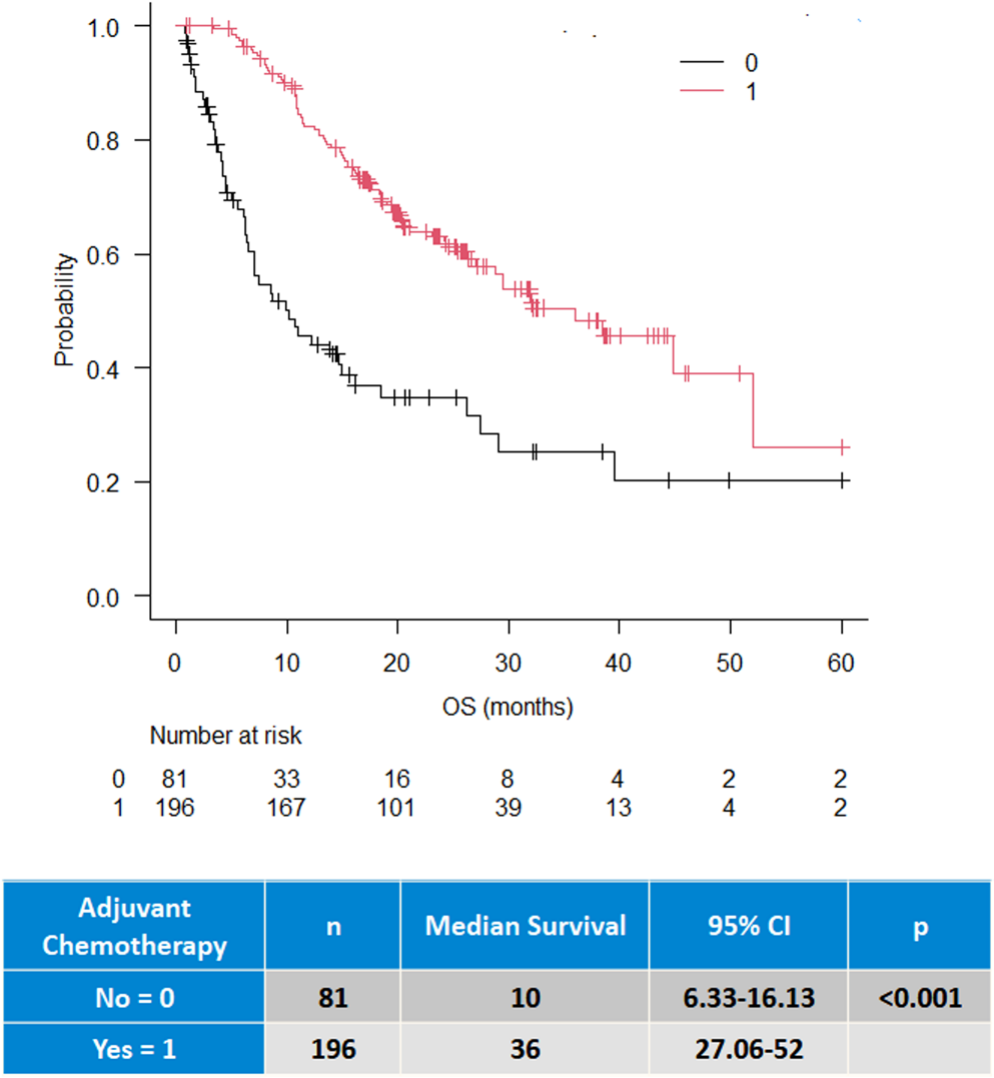
Overall survival with and without adjuvant chemotherapy administration

**Fig. 3:**
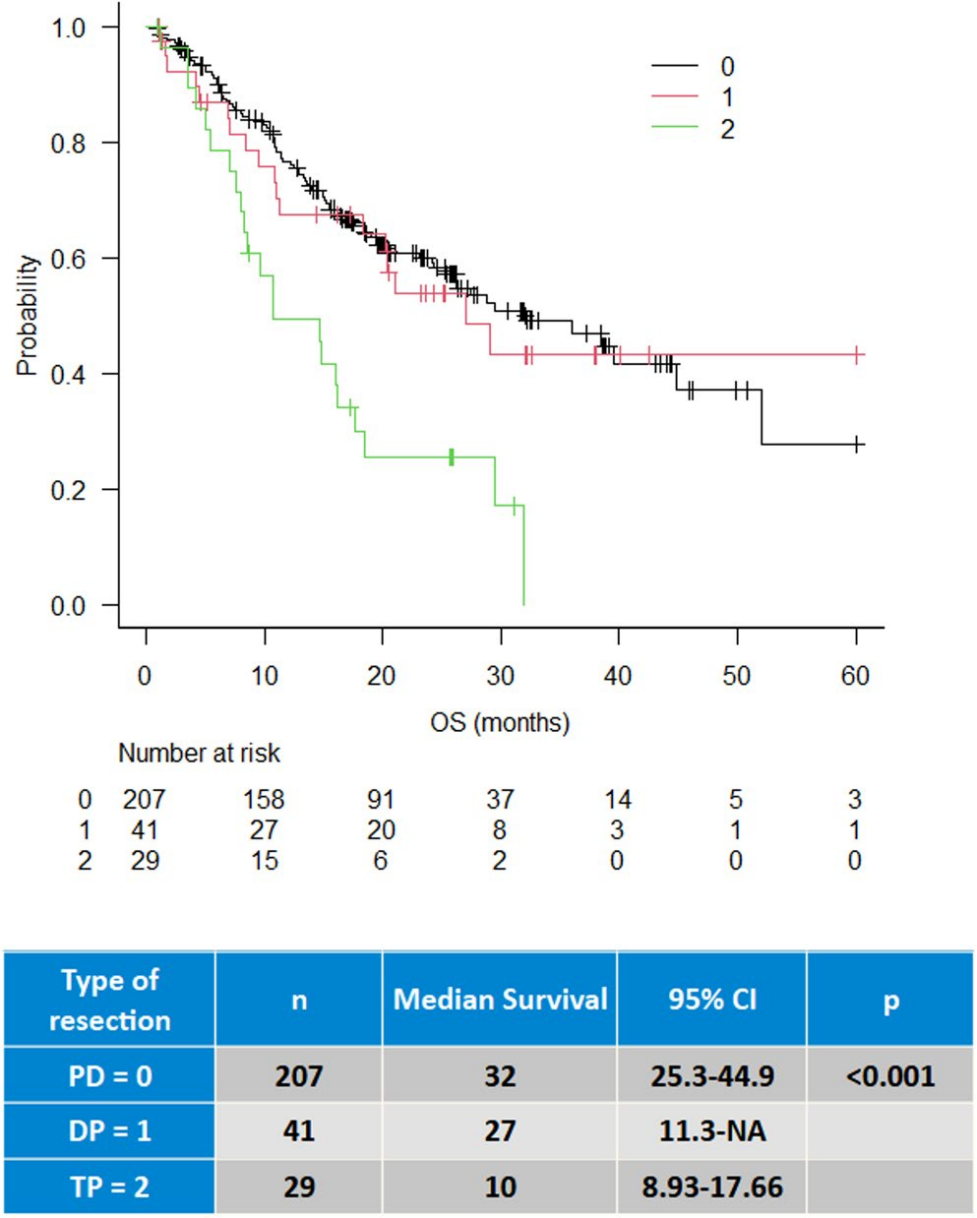
Overall survival according to type of resection

The median OS of the entire study cohort, except 3 patients lost at follow-up, was 27 months (20–36, 95% CI) (Fig. 1). Median OS of patients who received adjuvant therapy was 36 months (95% CI, 27–52), compared to 10 months in those who did not (*p* < 0.001) (Fig. 2). Regarding pancreatic resection type, the median OS was 32, 27, and 10 months for PD, DP, and TP, respectively (*p* < 0.001) (Fig. 3). No significant difference were observed in OS according to ISGPS type of venous resections although type IV showed the lowest survival (17 months) (Supplementary Fig. 1). The Cox regression multivariable analysis is presented in Table 4. Factors independently associated with survival included age ≥ 75 years (HR 2.4, 95% CI 1.9–4.4, *p* = 0.006), TP (HR 2.5, 95% CI 1.4–4.4, *p* < 0.001), N2 status (HR 2.1, 95% CI 1.3–3.2, *p* < 0.001), T1/T2 stage (HR 0.5, 95% CI 0.3–0.8, *p* = 0.008), and administration of adjuvant chemotherapy (HR 0.4, 95% CI 0.2–0.6, *p* < 0.001). Additional Cox regression model was done for the inclusion of POPF after excluding TP. None of the pancreatic-specific complications, regardless of grade, nor the occurrence of major morbidity were independently associated with poor survival.

**Table 4:** Univariable and multivariable Cox regression analysis of predictors for survival

Predictors	Univariable			Multivariable		
	HR	95% CI for HR	*P*	HR	95% CI for HR	*P*
Age >75 years	2.1	1.4–3.3	< 0.001	2.4	1.9–4.4	0.006
Gender Female	0.9	0.6–1.3	0.634			
ASA < 3	0.6	0.4–0.9	**0.011**	0.8	0.5–1.4	0.488
Surgery DP (ref.) vs. PD Total (ref.) vs. PD	1.1 2.6	0.6–1.8 1.6–4.3	0.695 **< 0.001**	2.5	1.4–4.4	**< 0.001**
Pretreatment Ca19-9 > 500	1.2	0.8–1.8	0.239			
Segmental vs. tangential resection Tangential	1	0.6–1.5	0.966			
Concomitant arterial resection yes	1.03	0.5–2.2	0.929			
Margin status R1	0.9	0.6–1.2	0.527			
pT T1-T2 vs. T3-T4	0.69	0.48–1	**0.053**	0.5	0.3–0.8	**0.008**
pN N0 N2	0.83 2.07	0.56–1.22 1.43–3	0.352 **< 0.001**	2.1	1.3–3.2	**< 0.001**
Relaparotomy yes	4.4	2.5–7.7	**< 0.001**	1.9	0.9–4.2	0.077
CR-POPF yes C	1.06 9	0.6–1.7 2.8–29	0.781 **< 0.001**	2.5	0.4–14.5	0.284*
PPH yes C	1.4 1.56	0.9–2.3 0.8–2.9	0.113 0.178			
DGE yes C	1.3 1.1	0.8–2 0.6–2.1	0.161 0.572			
Major morbidity yes	1.2	0.8–1.8	0.184			
Adjuvant chemotherapy yes	0.35	0.2–0.5	**< 0.001**	0.4	0.2–0.6	**< 0.001**

*PD* pancreatoduodenectomy, *DP* distal pancreatectomy, *TP* total pancreatectomy, *ISGPS* International Study Group on Pancreatic Surgery, *POPF* postoperative pancreatic fistula, *PPH* post-pancreatectomy hemorrhage, *DGE* delayed gastric emptying

*Included in other multivariate analysis without TP in which significant factors were Age > 75, N2, relaparotomy and adjuvant therapy administration.

## Discussion

The present study confirmed the safety and the acceptable oncologic outcomes of upfront pancreatectomy with venous resection for PDAC. The rate of complications was not different from that reported in literature regarding pancreatectomy without venous resection [14]. Despite the higher technical complexity due to venous resection, the survival outcomes of upfront PVR are not impaired when adjuvant chemotherapy is delivered. Adjuvant chemotherapy delivery is mostly dependent on preoperative patient’s performance status rather than pancreas-specific complications following PVR.

It is well established that pancreatic fistula is the main driver of morbidity after a pancreas resection. In the present study a 22% rate of POPF was observed, in line with that reported in literature [15]. Focusing on PVR, Hackert et al. reported a lower POPF rate in their series of 694 cases, with 4.6% for grade B and 1.2% for grade C [16]. However, such study had a different distribution of surgical procedures, including also resections after neoadjuvant, and the POPF grade C rates were similar. Therefore, the upfront setting might elevate the risk of POPF following a PVR without significantly impacting on the rate of severe events. Indeed, the 30- and 90-day mortality (3.5% and 6.3%, respectively) rates of the German study were also comparable to the present series. Another dreadful complication of PVR is represented by PPH, and in the present series this was observed in 15% of patients. The reported range of PPH in the literature varies from 3% to 28% [17]. Regardless of such relevant complications’ incidence, the current analysis of prognostic factors did not find any correlation between pancreas-specific complications and worse survival. This highlights the importance of mitigating the occurrence of pancreas specific complications, able to tune down their clinical significance. Moreover, it reveals the existence of other more relevant prognostic factors, particularly those related to the administration of adjuvant treatment and tumor biology. This finding is in line with other previous reports, including that of Crippa et al. in a large cohort of more than 2500 patients, where determinant factors of survival were related to tumor features and success of chemotherapy delivery [18].

The nature of the study does not allow for adding to the current debate regarding the ideal approach for localized, resectable/borderline PDAC. Several randomised control trials already exist, and others are in the pipeline [3, 19, 20]. However, common trends support the use of neoadjuvant treatment in patients with significant vessel involvement (despite technically “resectable”) to achieve a more radical surgery [3]. Another rationale for the systematic use of neoadjuvant therapy in PDAC lies in the assumption that patients are at high risk of complications and therefore may not receive adjuvant treatments [21]. In striking contrast with such knowledge, the present analysis suggests that postoperative access to adjuvant treatment is not negatively influenced by the burden of complications related to venous resection during pancreas surgery. Poor performance status emerged as the strongest independent limiting factor for adjuvant therapy. Other factors, including advanced age and major postoperative morbidity, may contribute, however, their independent impact is minor compared with the patient’s overall performance status, which remains the key determinant of chemotherapy eligibility even after upfront vascular resections. Of note, they also represent a limit for accessing neoadjuvant chemotherapy or for its actual completion in full scale [22]. The literature in fact attests that up to 25% of patients deemed fit for neoadjuvant chemotherapy are forced to interrupt it [23] and this holds true also in the setting of randomised clinical trials like the recently published NORPACT [24].

Closely related to the access to postoperative chemotherapy, the survival analysis of upfront PVR in the present series showed not inferiority compared to the benchmark outcomes for resected PDAC. Most patients (70%) indeed received adjuvant therapy, in line with a recently published series from the MGH [23], and their median OS was 36 months. These results should be compared to the 22 months OS reported in the literature [25], escalating up to 27 months when adjuvant chemotherapy is delivered [26]. Once again, other factors play a major role in determining survival after PVR, mainly those pertaining to the tumor biology and the patients’ performance status.

However, not all types of venous resection yield the same outcomes. ISGPS type IV resections, which involve graft interposition, were observed in only 7% of the series but were associated with higher rates of PPH, and a 90-day mortality of 14%. In their cohort of 694 PVR Hackert et al. reported a very low rate of type IV resections (4.7%) as well, again with a high incidence of pancreas-specific surgical morbidity [16]. Similarly, Groen et al. observed a 7.6% prevalence of type IV resections in a series of 351 patients, noting a higher 90-day mortality and morbidity when compared to other segmental resections [27]. Taken together, these findings suggest that, even in experienced hands, interposition grafts for PVR should be adopted carefully in the upfront setting, as the risk of graft thrombosis and severe postoperative complications is relevant. Another technical factor associated with poor survival in the present series was TP, as its median OS was only 10 months. TP for PDAC is mainly used for extended tumors of the pancreatic head/body, or once a positive resection margin is repeatedly found during a partial pancreatectomy. This approach has failed to prove an actual oncological benefit, as tumors not suitable for a R0 resection with a formal partial pancreatectomy are biologically more aggressive [28]. Another indication to perform a TP is the avoidance of a POPF once a high-risk anastomosis is required [29]. This is particularly felt as a priority once a complex vascular resection is also performed, to avoid the detrimental effect of an amylase rich fluid collection near a vascular reconstruction. However, a recent meta-analysis by Stoop et al. found no reduction in short-term mortality or major morbidity when TP was performed instead of PD in high-risk patients, although data on long-term OS were not provided [30]. These results suggest that TP done for oncologic or vascular reasons should be avoided in an upfront setting and should not be routinely performed to prevent POPF and its complications, but carefully considered only as a bail-out procedure.

Our study highlights that patients classified as resectable, or even borderline resectable but unsuitable for chemotherapy, may still be considered for upfront surgery when Type I–III venous resection is required, as postoperative morbidity remains acceptable and does not prevent subsequent adjuvant treatment, provided the procedure is performed in high-volume, experienced centers. On the other hand, in patients presenting with intraoperative evidence of a significantly more advanced disease compared to preoperative imaging (e.g., invasion of the porto-mesenteric axis requiring graft interposition and/or total pancreatectomy) resection should be aborted, and the patient referred to neoadjuvant therapy.

The limitations of the present study include its retrospective nature, particularly regarding patient selection and potential misclassification during the study period. The study population was drawn from two different continents, where recognized differences exist in medical practice, physical factors, surgical approaches, and preferences regarding venous resections and types of reconstruction. The surgical departments of the two centers involved are, however, highly specialized in pancreatic surgery with extensive expertise in vascular reconstruction and access to all types of grafts. Additionally, both centers have a long-standing experience in optimizing surgical outcomes, specifically aimed at reducing the incidence and severity of POPF.

Despite the progress made and the evidence presented, several uncertainties remain, necessitating further research into potential future developments. Two major issues require in-depth investigation. The first concerns the optimal sequencing of chemotherapy and surgery, given the comparable outcomes of PVR. Determining the most effective sequence for patients with PDAC and vascular involvement is crucial to ensure the best survival outcomes while minimizing treatment-related morbidity. The second key issue is the correlation between preoperative radiological findings, the actual vascular involvement from the tumor encountered during surgery, and the histopathological confirmation of the venous invasion. Addressing this question is essential to refining vessel-sparing surgical strategies and improving patient selection for vascular resection.

## Conclusion

PVR should be considered in patients with PDAC also in an upfront setting at high volume centers, given its satisfactory surgical outcomes without decreased chances to proceed with adjuvant chemotherapy. Both OS and the administration of adjuvant therapy appear to be not significantly affected by pancreas-specific complications but are instead influenced by the tumor biology and patient performance status. ISGPS type IV resections and total pancreatectomy should be discouraged in this setting as they are associated with high perioperative morbidity and mortality. Further studies are needed to confirm these findings and to determine the optimal treatment strategy for localized, resectable PDAC eventually found to have a vascular involvement.

## Supplementary Information

Below is the link to the electronic supplementary material.

Supplementary file1 DOCX (10.4 KB)

Supplementary file2

## Data Availability

The data supporting this study are stored in a secure database at the promoting institution and can not be made publicly available due to privacy restrictions. De-identified data may be made available upon reasonable and well-justified written request to the corresponding author.
