# Supplementary material for: Complications following upfront pancreatectomy with venous resection do not compromise adjuvant chemotherapy delivery and survival in pancreatic cancer
Source: Langenbecks Arch Surg. 2025 Nov 27;411(1):37. doi: 10.1007/s00423-025-03933-z (PMC12743065; doi:10.1007/s00423-025-03933-z)
Supplement: Supplementary file 1 — DOCX (10.4 KB) [file 423_2025_3933_MOESM1_ESM.docx]

| **Supplementary Table 1. Complications & mortality rates according to ISGPS types of venous resection** | | | | | | |
| --- | --- | --- | --- | --- | --- | --- |
| **Events** | **Total, No. (%) (N= 280)** | **ISGPS Type, No. (%)** | | | | ***P*** |
|  |  | I.(Tangential: primary closure)  (N= 49) (18%) | II.(Tangential: peritoneal patch)  (N= 21) (8%) | III.(Segmental: primary anastomosis) (N=189) (66%) | IV. (Segmental: interposition graft)  (N=21) (8%) |  |
| Surgery type, No. (%)  PD  DP  TP | 210 (79%)  41 (15%)  29 (10%) | 29 (59%)  16 (33%)  4 (8%) | 16 (76%)  3 (15%)  2 (9%) | 150 (79%)  20 (11%)  19 (10%) | 15 (72%)  2 (9%)  4 (19%) | **0.008** |
| DGE (yes)  Grade:  A  B  C | 51 (18%)  15 (5%)  13 (5%)  23 (8%) | 10 (20%)  4 (8%)  2 (4%)  4 (8%) | 6 (28%)  2 (9%)  2 (9%)  2 (9%) | 32 (17%)  8 (4%)  8 (4%)  16 (8%) | 3 (14%)  1 (5%)  1 (5%)  1 (5%) | 0.553  0.925 |
| PPH (yes)  Grade:  A  B  C | 43 (15%)  12 (4%)  11 (4%)  20 (7%) | 6 (12%)  1 (2%)  0  5 (10%) | 6 (28%)  2 (9%)  2 (9%)  2 (9%) | 24 (13%)  4 (2%)  9 (5%)  11 (6%) | 7 (33%)  5 (24%)  0  2 (10%) | **0.024**  **<0.001** |
| POPF (yes)  Grade:  B  C | 56 (22%)  53 (21%)  3 (1%) | 16 (35%)  14 (31%)  2 (4%) | 7 (37%)  7 (37%)  0 | 31 (18%)  30 (17.5%)  1 (0.5%) | 2 (12%)  2 (12%)  0 | **0.022**  **0.041** |
| Major morbidity | 96 (34%) | 20 (41%) | 10 (48%) | 57 (30%) | 9 (43%) | 0.195 |
| Relaparotomy | 23 (8%) | 7 (14%) | 3 (14%) | 10 (5%) | 3 (14%) | 0.086 |
| Mortality 30-day | 3 (1%) | 1 (2%) | 0 | 1 (0.5%) | 1 (5%) | 0.274 |
| Mortality 90-day | 12 (4%) | 4 (8%) | 0 | 5 (3%) | 3 (14%) | **0.028** |
| Adjuvant chemotherapy administration | 196 (70%) | 31 (63%) | 13 (62%) | 139 (73%) | 13 (62%) | 0.321 |
| *Abbreviations: PD, pancreatoduodenectomy; DP, distal pancreatectomy; TP, total pancreatectomy; ISGPS, International Study Group on Pancreatic Surgery; DGE, delayed gastric emptying; PPH, post-pancreatectomy hemorrhage; POPF, postoperative pancreatic fistula* | | | | | | |

| **Supplementary Table 2. Complications & mortality rates according to types of resection** | | | | | |
| --- | --- | --- | --- | --- | --- |
| **Events** | **Total, No. (%) (N= 280)** | **Resection type, No. (%)** | | | ***P*** |
|  |  | PD  (N=210) (75%) | DP  (N=41) (15%) | TP  (N=29) (10%) |  |
| ISGPS venous type  I  II  III  IV | 49 (18%)  21 (7%)  189 (68%)  21 (7%) | 29 (14%)  16 (8%)  150 (71%)  15 (7%) | 16 (39%)  3 (7%)  20 (49%)  2 (5%) | 4 (14%)  2 (7%)  19 (65%)  4 (14%) | **0.008** |
| DGE (yes)  Grade:  A  B  C | 51 (18%)  15 (5%)  13 (5%)  23 (8%) | 39 (18%)  11 (5%)  11 (5%)  17 (8%) | 6 (15%)  2 (5%)  0  4 (10%) | 6 (21%)  2 (7%)  2 (7%)  2 (7%) | 0.783  0.833 |
| PPH (yes)  Grade:  A  B  C | 43 (15%)  12 (4%)  11 (4%)  20 (7%) | 30 (14%)  9 (4%)  8 (4%)  13 (6%) | 4 (9%)  1 (2%)  0  3 (7%) | 9 (31%)  2 (7%)  3 (10%)  4 (14%) | **0.035**  0.202 |
| POPF (yes)  Grade:  B  C | 56 (22%)  53 (21%)  3 (1%) | 36 (17%)  33 (16%)  3 (1%) | 20 (49%)  20 (49%)  0 |  | **<0.001**  **<0.001** |
| Major morbidity | 96 (34%) | 63 (30%) | 23 (56%) | 10 (34%) | **0.006** |
| Relaparotomy | 23 (8%) | 15 (7%) | 3 (7%) | 5 (17%) | 0.174 |
| Mortality 30-day | 3 (1%) | 3 (1%) | 0 | 0 | 0.603 |
| Mortality 90-day | 12 (4%) | 8 (4%) | 3 (7%) | 1 (3%) | 0.582 |
| Adjuvant chemotherapy administration | 196 (70%) | 151 (72%) | 27 (66%) | 18 (62%) | 0.457 |
| *Abbreviations: PD, pancreatoduodenectomy; DP, distal pancreatectomy; TP, total pancreatectomy; ISGPS, International Study Group on Pancreatic Surgery; DGE, delayed gastric emptying; PPH, post-pancreatectomy hemorrhage; POPF, postoperative pancreatic fistula* | | | | | |

**Supplementary Figure 1.** Overall Survival according to ISGPS type of venous resections
