# Supplementary figures and images for: Complications following upfront pancreatectomy with venous resection do not compromise adjuvant chemotherapy delivery and survival in pancreatic cancer
Source: Langenbecks Arch Surg. 2025 Nov 27;411(1):37. doi: 10.1007/s00423-025-03933-z (PMC12743065; doi:10.1007/s00423-025-03933-z)

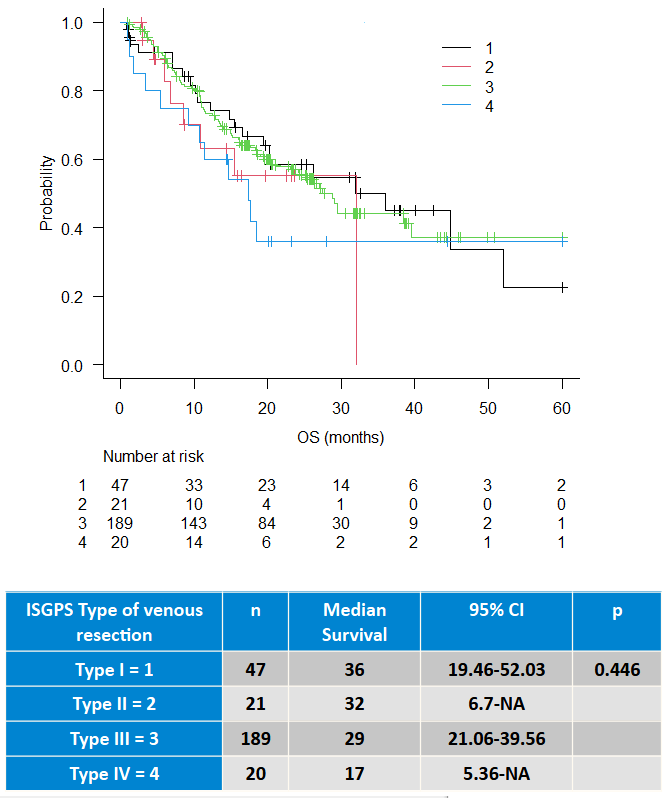

Supplement: Supplementary file 2 — Supplementary file2 [file 423_2025_3933_MOESM2_ESM.png]
